# Fourteen years of manifestations and factors of health insurance fraud, 2006–2020: a scoping review

**DOI:** 10.1186/s40352-021-00149-3

**Published:** 2021-09-30

**Authors:** José Villegas-Ortega, Luciana Bellido-Boza, David Mauricio

**Affiliations:** 1 Universidad Nacional Mayor de San Marcos, Av. Germán Amezaga 375, 15081 Lima, Peru; 2 Universidad Escuela Superior de Administración y Negocios, Lima, Peru; 3 Universidad Peruana de Ciencias Aplicadas, Facultad de Ciencias de la Salud, Lima, Peru

**Keywords:** Healthcare, Fraud, Insurance, Behaviour, Factor, Manifestation

## Abstract

**Background:**

Healthcare fraud entails great financial and human losses; however, there is no consensus regarding its definition, nor is there an inventory of its manifestations and factors. The objective is to identify the definition, manifestations and factors that influence health insurance fraud (HIF).

**Methods:**

A scoping review on health insurance fraud published between 2006 and 2020 was conducted in ACM, EconPapers, PubMed, ScienceDirect, Scopus, Springer and WoS.

**Results:**

Sixty-seven studies were included, from which we identified 6 definitions, 22 manifestations (13 by the medical provider, 7 by the beneficiary and, 2 by the insurance company) and 47 factors (6 macroenvironmental, 15 mesoenvironmental, 20 microenvironmental, and 6 combined) associated with health insurance fraud. We recognized the elements of fraud and its dependence on the legal framework and health coverage. From this analysis, we propose the following definition: “Health insurance fraud is an act of deception or intentional misrepresentation to obtain illegal benefits concerning the coverage provided by a health insurance company”. Among the most relevant manifestations perpetuated by the provider are phantom billing, falsification of documents, and overutilization of services; the subscribers are identity fraud, misrepresentation of coverage and alteration of documents; and those perpetrated by the insurance company are false declarations of benefits and falsification of reimbursements. Of the 47 factors, 25 showed an experimental influence, including three in the macroenvironment: culture, regulations, and geography; five in the mesoenvironment: characteristics of provider, management policy, reputation, professional role and auditing; 12 in the microenvironment: sex, race, condition of insurance, language, treatments, chronic disease, future risk of disease, medications, morale, inequity, coinsurance, and the decisions of the claims-adjusters; and five combined factors: the relationships between beneficiary-provider, provider-insurance company, beneficiary-insurance company, managers and guānxi.

**Conclusions:**

The multifactorial nature of HIF and the characteristics of its manifestations depend on its definition; Identifying the influence of the factors will support subsequent attempts to combat HIF.

**Supplementary Information:**

The online version contains supplementary material available at 10.1186/s40352-021-00149-3.

## Background

Corruption and fraud are embedded in health systems (HS), and they are motivated by abuse of power and dishonesty (García, 2019) that harm the user population, generating economic and even human losses (World Bank, 2018). The different aspects of corruption seriously weaken the access and performance of the HS; among the most affected, equity, quality, response capacity, efficiency and resilience should be mentioned (W. H. Organization, 2016). In the world, more than seven billion dollars are spent on health services from those,  between 10% and 25% of spending is directly lost as a result of corruption, an amount that exceeds the annual estimate for 2030 in providing universal health coverage (Jones & Jing, 2011; World Bank, 2019). In addition, there is a constant increase in healthcare spending, healthcare professionals seeking to maximize their profits, and health insurance seeking to contain costs (Dumitru et al., 2011; Wan & Shasky, 2012).

Fraud in the HS is often included in the discussion of corruption since these practices generally involve abuse of power (Vian, 2020). Health insurance fraud (HIF) is a substantive component of the HS crisis (Manchikanti & Hirsch, 2009). The HIF mainly affects developing countries with fewer resources (Perez & Wing, 2019), weakened health systems and a lack of quality, causing significant losts and inefficiencies (Kruk et al., 2018). Losses caused by HIF in some high-income countries range between 3 and 10% (Rashidian et al., 2012), and its main motivation is the search for money by fraudsters, to which other individuals, organizational or contextual factors are added (Busch, 2012; Wan & Shasky, 2012). HIF is a problem that ranks second after violent crimes in the United States (USA) (Sparrow, 2008) and can be committed by medical providers, policyholders and health insurers (Busch, 2008). In this sense, it is essential to identify and understand the factors that influence HIF and its manifestations to combat them and reduce losses in HS.

The public health programmes of the different countries of the world propose interventions to prevent and detect HIF, many of which lack effectiveness in their results. Although the interventions include multiple deterrence efforts and strategies based on data mining, they are insufficient to show effective results to combat HIF (Abdallah et al., 2016; Bayerstadler et al., 2016; Hassan & Abraham, 2013; Joudaki et al., 2015; Kang et al., 2010; Kelley et al., 2015; Kose et al., 2015; Li et al., 2008; Lin et al., 2013; Ormerod et al., 2012; Perez & Wing, 2019; Rashidian et al., 2012; Shi et al., 2016). Likewise, scientific evidence indicates a shortage  of studies that address how to deal with fraud effectively in the health sector; however, it identifies some promising interventions, such as the actions of an independent agency, prohibitions, internal control practices, transparency, accountability, among others, but it is unknown whether or not they contribute to reducing corruption (Gaitonde et al., 2016). In this same sense, the evidence shows that despite the efforts made to reduce HIF, it is a complex problem difficult to address.

HIF, as a fraud, is multifaceted, multidimensional and interrelated, mainly caused by the insufficiency of theories that can explain its complexity (Huber, 2017). Part of the complexity of the HIF is supported by the dynamic behaviour of fraudsters, which generates the need for human interaction to identify suspected cases (Travaille et al., 2011), given the scarce specialization in detection interventions, which are limited to opportunistic verifications of previously known patterns and detections by coincidence (Bayerstadler et al., 2016). On the other hand, complexity is supported by the lack of a standardized definition of the HIF, which does not have a consensual definition; however, it could refer to deception or intentional misrepresentation used to obtain illegal benefits, making it difficult to distinguish from abuse, waste or error (Hyman, 2001; Joudaki et al., 2015; Lee et al., 2020; Rashidian et al., 2012) In addition, its manifestation will depend on regulation and market behaviour (Bayerstadler et al., 2016; Green, 2007). The proposal of a definition seeks to contribute to the development of better strategies (Kacem et al., 2019).

Given the absence of a standardized definition of HIF, this scoping review could contribute to filling a gap in knowledge, providing a definition with a homogeneous language, which can dispel ambiguity and facilitate its understanding. In this sense, the objective of our scope review is to define the HIF, identify the causes or factors that influence, and the consequences or manifestations that occur; for which we will answer the following questions: What is health insurance fraud? How is health insurance fraud manifested? Furthermore, what factors influence health insurance fraud?

The results obtained are intended to promote future studies that more effectively channel the interventions that prevent, detect, and provide responses to combat HIF and be a reference for decision-making in countries’ public health.

## Methods

To answer our research questions, we conducted a scoping review, using a rigorous literature review method, which establishes conceptual limits, following the considerations of the document “PRISMA Extension for Scoping Reviews (PRISMA-ScR): Checklist and Explanation” (Tricco et al., 2018). In Additional file 1, we include the Checklist for Scoping Reviews (PRISMA-ScR).

Scoping reviews allow answering broad questions such as those posed by our study, while systematic reviews allow answering clearly defined questions (Tricco et al., 2018). The scoping review aims to identify gaps in knowledge, analyze the literature body, clarify concepts, or investigate the nature of a problem (Munn et al., 2018). In this sense, our research questions seek to examine and clarify the definition used in the literature on HIF and show how this theoretical definition becomes tangible and shown in reality through the manifestation. As fraud is a complex problem, we also seek to identify and inventory the associated factors that influence it. Our contribution provides new elements of judgment to confront the HIF, it will facilitate the redesign and innovation of practical strategies that help combat fraud, and we hope that other studies will join the few that have demonstrated effectiveness in their interventions.

### Eligibility criteria

We reviewed studies according to our objective, and we included those reviewed by peers and are indexed to international databases since they are considered validated knowledge. We limited our search from January 1, 2006, to July 31, 2020, taking into account that the U.S. Department of Health and Human Services issued in 2006, the final rule that establishes civil monetary sanctions, procedures for investigations and hearings, for violating the Health Information Portability and Privacy Act (Tovino, n.d.). In addition, we observed that starting in 2006, monetary recoveries from HIF sanctions increased by 40% compared to the previous 20 years (Helmer Jr, 2012). The search had no geographic or language restrictions. However, we exclude conferences, proceedings, posters, editorials, letters, misprints and books, as they do not provide reliable scientific evidence. We also excluded studies with meanings ascribed to health abuse related to drugs, diseases, suicide, racism, food fraud, security, network, web, and  electronics device; all of them for being away from the health insurance environment.

### Information sources

To identify the documents, we conducted a scoping review search in ACM, EconPapers, PubMed, Science Direct, Scopus, Springer and Web of Science between January 1, 2006, and July 31, 2020.

### Search strategies

To guarantee not to lose potential studies, we applied an iterative approach; we initially used the studies that met the inclusion criteria in PubMed and WoS. The search strategy included a combination of keywords and medical topic headings (MeSH for PubMed), terms related to “Fraud healthcare” (concept A) and “Health Insurance” (concept B). Subsequently, we used the results of this search to identify keywords and MeSH terms, which were adapted in the search strategies of the other databases, as reported in Additional file 2. A health librarian reviewed the search strategy.

### Data process, evaluation and quality of the study

Once we applied the search strategies and identified the potential studies, two authors made the review separately as follows: we designed a matrix in Excel, in which we listed all the studies characterized by name, code, among others (Additional file 3). In order to perform the first screening in the database, we ordered the studies by titles and authors, and eliminated duplicates, both authors in consensus. In a second selection, we reviewed the titles, abstracts and conclusions of the selected studies; for this, each author considered the eligibility criteria of the study, in the end, both authors agreed on the list. Once the possible studies had been chosen, the two authors read the full text, which allowed us to select those that contribute directly to the research questions (Theoretical Fraud, Practical Manifestation, Factors), and we identified them in the matrix; finally, the authors reached at a consensus. Subsequently, we evaluated the content and explicit references to answer the research questions; We have avoided conjectures or interpretations. In addition, we considered the theoretical or experimental contribution of each study for the factor inventory and showed the influence (if the factor increases or worsens the HIF, we used the positive sign, while if the factor reduces the HIS, we registered the negative sign; a result could also indicate an ambivalent influence so we used both signs). We have considered a consensus greater than 95%, and the differences were discussed and cleared for both autor based on screening, eligibility, and final inclusion of the studies. However, we excluded studies that did not contribute to any questions.

We reviewed 67 pairs of articles included in the study, both qualitative and quantitative, and with the help of the matrix, we discussed the results and reached a consensus on the information extraction. To evaluate the quality and rigour of the studies, we used a tool for integrative reviews, which is based on four factors: type of study, sampling method, detail of the data collection method, and analysis. The possible score generated by this tool varies between 4 (qualitative design, sampling and collection of unexplained data, and narrative analysis) and 13 (quantitative experimental design, random sampling, explained data collection and inferential statistics) (Olsen & Baisch, 2014; Pfaff et al., 2014). The details of the quality scores of the included studies can be seen in Additional file 4. To ensure the strength of the evidence and the studies’ quality, the authors independently graded the articles in rounds, and disagreements were resolved through discussion until an agreement more significant than 95% was achieved. In addition, we evaluated the SCImago journal rank (SJR), which measures the scientific influence of academic journals according to the number of citations of the journal to which each of the included manuscripts belonged, which is justified by the high quality of publications over the years (Ardito et al., 2015).

## Results

### Selection of studies

**Fig. 1:**
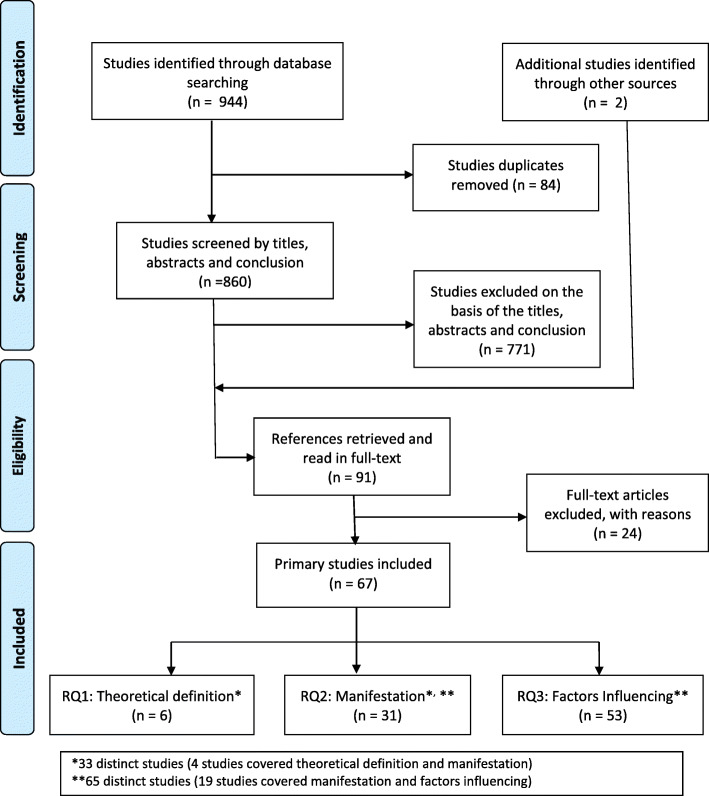
PRISMA flow diagram

A total of 944 studies were identified following the selection criteria. Subsequently, 84 duplicate studies were eliminated, and then the titles, abstracts and conclusions of 860 studies were examined, from which 89 full texts were recovered, to which we incorporated two relevant studies identified from other sources (a thesis referred by the authors selected from 2003 and another reference document on fraud issues of the National Health Care Anti-Fraud Association of 2018). In the process, we excluded 24 studies related to HIF detection techniques, data mining models, processes, activities or other aspects not related to the factors and manifestations of HIF. Finally, we included 67 studies (Fig. 1).

### RQ1: definition of health insurance fraud

We identified six definitions of HIF and the key elements of each of them to integrate them into a single consensual definition, as shown in Table 1. General definitions of fraud that included intensity of desire, risk of apprehension, violation of trust, rationalization (Ramamoorti & Olsen, 2007), also defined as the obtaining of a financial advantage, or the cause of a loss through an implicit or explicit deception using a mechanism through which the fraudster obtains an illegal advantage or causes an unlawful loss (Levi & Burrows, 2008).

**Table 1:** Definitions of health insurance fraud

Definition	Studies	Key elements identified^a^				
		A	B	C	D	E
Deception or intentional misrepresentation that the person or entity makes knowing that the misrepresentation could result in an unauthorized benefit for the person, entity, or another part.	(NHCAA, 2018)	X	X	X		
Criminal act as a violation of civil law according to the law. Behaviours are ranging from intentional misrepresentation of services provided to inadequate documentation for Medicare/Medicaid.	(Gasquoine & Jordan, 2009)	X	X		X	X
Abuse of the system of a for-profit organization without necessarily having direct legal consequences, while prescription fraud is defined as the illegal acquisition of prescription drugs for personal use or profit and could be observed in many ways.	(Aral et al., 2012)			X		X
Deception or intentional misrepresentation used to obtain illegal benefits.	(Joudaki et al., 2015)	X	X	X		
A severe federal crime and includes filing claims with the intention of “defrauding”.	(Dolan & Farmer, 2016)	X	X		X	
Any activity with malicious intent resulting in personal benefit.	(Sheffali & Deepa, 2019)	X	X	X		
Total	6	5	5	4	2	2

^a^ Key elements identified:

A. Deception or misrepresentation/Behaviours

B. Intentional

C. Unauthorized benefit/for-profit/personal benefit

D. Criminal act according to the law/serious federal crime

E. Health insurance/System abuse

Based on what is described in Table 1, we have identified and classified five key elements that incorporate the six definitions shown, and subsequently, we have integrated them, as shown below:

A. It is deceptive, and the people involved tend to deceive, lie, hide and manipulate the truth. Five definitions affect the term “deception”, which is associated with an act linked to misrepresentation and deception.
B. It is intentional; Fraud is not the result of simple error or negligence but involves deliberate attempts to obtain an illegal advantage; thus, it induces a course of action predetermined by the perpetrator (Pickett & Pickett, 2002) 47. Five definitions affect the term “intentional”, which is associated with a deliberate act.
C. Obtains a benefit, profit or advantage; Usually, the benefit is economic, which implies that there is a victim and that the action produces losses of individual, organizational, and even national resources.
D. It is illegal, and some definitions describe it as a criminal act or severe federal crime. To establish an illegal act, you must break the law. Some practices may be legal in some countries, but not necessarily in others; it will depend on the rules and regulations of each country or state.
E. Health insurance coverage (HIC), taken from the definition of health insurance, allows us to circumscribe the scope. The synonyms of HIC used are “health coverage”, “medical care coverage”, and “health benefits” (Elwyn et al., 2000) 48.

To have a single definition of HIF, we have again integrated what is described in A, B, C and D, from which we obtained that “Fraud is a deliberate deception to obtain unfair or illegal profits”, a statement that is complemented by described in E. We specified that the absence of one of five key elements identified puts at risk the comprehensive definition of HIF. Finally, we arrived at the following definition:

“Health insurance fraud is an act based on deceit or intentional misrepresentation to obtain illegal benefits concerning the coverage provided by health insurance.”

**Fig. 2:**
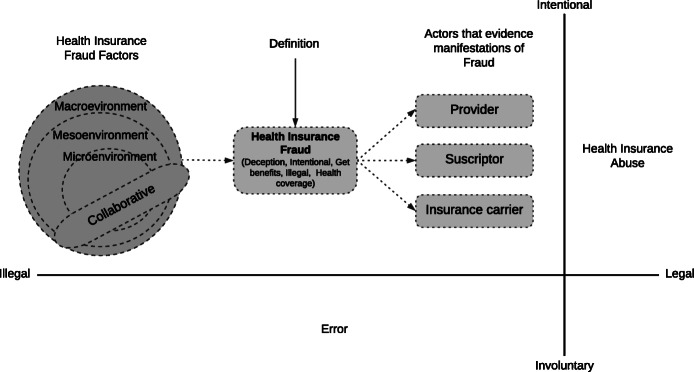
Elements of the definition of health insurance fraud, its factors and manifestations

To illustrate the elements that comprise the definition of HIF, we showed Fig. 2, in which we show the relationship they have with its factors (RQ2) and manifestations (RQ3).

### RQ2: manifestations of health insurance fraud

We found that fraud manifests itself in multiple ways, such as performing unnecessary services, falsifying records, separating invoices, and misrepresented coding. Therefore, we classified the manifestations by actor (Li et al., 2008; Sheffali & Deepa, 2019). In this sense, we present the manifestations by a) health service providers, including hospitals, laboratories, and health care professionals; b) insurance underwriters, including patients; and c) public or private health insurance companies, which include state-subsidized programmes.

In 23 studies, we identified 13 manifestations of fraud by the provider; the three most common manifestations are phantom billing based on claims presented for medical services not provided, mentioned in 14 studies, the falsification of administrative or clinical documents in 10 studies and the proportion of unnecessary care in 9 studies. In 16 studies, we identified seven manifestations of fraud by the subscriber; the three most frequent are identity fraud mentioned in 8 studies, manipulating the eligibility information and manipulation of documents, both noted in 4 studies. Finally, in two of the studies, two manifestations of fraud of the agent or insurer were identified: false declarations of benefits or services and falsification of reimbursements. Each manifestation was briefly described, and one or more examples were provided. We observe that each study could contribute to more than one manifestation. For a better understanding, we showed Table 2.

**Table 2:** Manifestation of health insurance fraud by the provider

Manifestation	Description (examples)	Studies
By the provider		
Self -referral	A scheme for recommending patients to their own or third-party provider has a financial relationship with the originator of the referral. ^(a)^	(Francis, 2020)
Upcoding	Intentionally coding a health claim based on an inaccurate use of codes to obtain greater economic value. ^(b)^	(Gasquoine & Jordan, 2009; Massi et al., 2020; Palutturi et al., 2019; Phillipsen et al., 2008; Sheffali & Deepa, 2019)
Unperformed or billing for services not provided	Known as phantom billing, claims are presented for medical services, medications, medical devices not delivered to the patient.^(c)^	(Aral et al., 2012; Bauder & Khoshgoftaar, 2020; Bayerstadler et al., 2016; Brooks et al., 2012; Dolan & Farmer, 2016; Gasquoine & Jordan, 2009; Jou & Hebenton, 2007; Lee et al., 2016; Li et al., 2008; Palutturi et al., 2019; Perez & Wing, 2019; Phillipsen et al., 2008; Smith et al., 2013; Yang, 2003)
Misrepresenting the diagnosis or procedure to justify payment	Manipulation of procedures, diagnoses, requests, complaints, dates, frequency, duration or description of the services provided. ^(d)^	(Gasquoine & Jordan, 2009; Li et al., 2008; Phillipsen et al., 2008; Shin et al., 2012; Yang, 2003)
Soliciting, offering, or receiving a kickback	A bribe is defined as a financial or other advantage offered, granted, requested or accepted in exchange for privileges or treatment.^(e)^	(Gasquoine & Jordan, 2009; Perez & Wing, 2019; Sheffali & Deepa, 2019)
Unbundling or *exploding* charges (bundled services are supposed to be paid at a group rate)	Creating separate claims for services or supplies that should be grouped. It can be seen as part of an incorrect codification, but several authors mention it as a separate form of fraud. ^(f)^	(Bayerstadler et al., 2016; Gasquoine & Jordan, 2009; Li et al., 2008; Manocchia et al., 2012; Palutturi et al., 2019; Perez & Wing, 2019; Phillipsen et al., 2008; Shin et al., 2012)
Falsifying certificates of medical, plans of treatment, medical records.	It is manipulating documents (clinical history, invoice, clinical exams, prescriptions or certificates), prices, and services to achieve economic benefit. ^(g)^	(Dolan & Farmer, 2016; Gasquoine & Jordan, 2009; Jou & Hebenton, 2007; Li et al., 2008; Lin et al., 2009; Manocchia et al., 2012; Phillipsen et al., 2008; Sheffali & Deepa, 2019; Victorri-Vigneau et al., 2009; Yang, 2003)
Unjustified services, Overutilisation, Providing unnecessary care	Providing unnecessary medical care or Billing more expensive procedures or services. ^(h)^	(Bayerstadler et al., 2016; Dolan & Farmer, 2016; Francis, 2020; Jou & Hebenton, 2007; Li et al., 2008; Palutturi et al., 2019; Perez & Wing, 2019; Wan & Shasky, 2012; Yang, 2003)
Opportunistic fraud,	Billing for services provided by unqualified personnel without credentials or licence to give that type of care. ^(i)^	(Aral et al., 2012; Phillipsen et al., 2008; Weiss et al., 2015)
Repeat billing or billing twice for the same service provided	Charging more than once for the same procedure, medications and medical devices, even if they are only administered once. ^(j)^	(Dolan & Farmer, 2016)
Readmission or admission	Readmissions apply to hospitalized patients who require prolonged treatment (administratively discharge), dividing them into two episodes when they are not discharged. ^(k)^	(Palutturi et al., 2019)
Type of room Charge	Billing the cost of care for a room whose treatment class is higher than the one used by the patient. ^(l)^	(Palutturi et al., 2019)
Cancelled services, Underbilling or “write-offs” such as professional discounts and courtesies.	Involve the billing of medications, procedures or services previously planned but then cancelled, includes billing of discounts and professional courtesies provided. ^(m)^	(Dolan & Farmer, 2016; Palutturi et al., 2019)
By the subscriber		
Using the wrong diagnosis to justify payment	Medical reimbursements are sent by filling out claim forms for a service provided based on a diagnosis. These diagnoses can also be manipulated. ^(n)^	(Shin et al., 2012)
Price and documents manipulation	It is manipulating documents (clinical exams, certificates, medical prescription, among others) to achieve an economic benefit. ^(o)^	(Bayerstadler et al., 2016; Dolan & Farmer, 2016; Lin et al., 2009; Manocchia et al., 2012)
Unperformed billing for services not provided	Patients file false claims alone or in collusion with friends or healthcare professionals to collect fraudulent medical reimbursement. ^(p)^	(Ekin et al., 2018;Li et al., 2008 ; Yang, 2003)
Opportunistic Fraud	It is a case of opportunistic or occasional fraud, one in which “the reality of a claim is taken advantage of to introduce pre-existing or previous damages”. ^(q)^	(Ribeiro et al., 2020; Zhou et al., 2016)
Identity fraud included using ghost or deceased employees.	Obtain and use someone else’s health insurance card to get health care or other services. This situation can occur with or without the knowledge of the owner. ^(r)^	(Baltussen et al., 2006; Dolan & Farmer, 2016; Goel, 2020; Jator & Hughley, 2014; Johnson & Nagarur, 2016; Li et al., 2008; Sheffali & Deepa, 2019; Shin et al., 2012)
Doctor shopping	Patients seek to stock up on controlled substances or drugs. ^(s)^	(Sheffali & Deepa, 2019)
Misrepresenting eligibility	Patients can misrepresent information about themselves or their dependents to obtain medical coverage, which is not eligible. ^(t)^	(Geruso & Rosen, 2015; Li et al., 2008; Sheffali & Deepa, 2019; Yang, 2003)
By the insurer company		
Falsifying benefit or service statements	Agent or insurer who falsifies statements of benefits or services. ^(u)^	(Li et al., 2008; Yang, 2003)
Falsifying reimbursements	Agent or insurer falsifying reimbursements. ^(v)^	(Li et al., 2008; Yang, 2003)

^a)^ Refer patients to a clinic, diagnostic service, hospital, among others, with whom they have an economic relationship; if the referred pays a commission, a bribe could be configured (Thornton et al., 2015)

^b)^ One nurse coded CPT 99212 “problem-focused office visit for a patient” with no history or physical exams in the medical record; she just got a tetanus booster. The coding identified the patient with back pain, which the patient denied, a false diagnosis coding was identified (Phillipsen et al., 2008)

^c)^ The insurance company received a bill for USD 600 for CPT Code 93980 (for the penile duplex scan), USD 300 for CPT code 54240 (penile plethysmography), USD 95 for CPT code 59504 (for a nerve conduction study) and USD 165 for CPT code 99214 E&M (no history or detailed exam). The medical file contained a “Vascular Profile for Free Diagnostic Evaluation” sheet signed by the professional, which consisted of a vascular profile and biotelemetry of the penis (Barrett, 2006)

^d)^ A patient visit before a planned vasectomy was billed as a CPT 99245 (‘level 5’) office visit that included a complete history, comprehensive examination, highly complex medical decision making, even though there was no blood pressure, height, weight, pulse or breaths in the medical record. At best, the registry supports a preoperative vasectomy visit focused on CPT 99241 (‘level 1’) problem. In addition, the procedure fee includes a preoperative visit (Phillipsen et al., 2008)

^e)^ Pharmacists may fill a prescription with a specific brand of drugs rather than another that yields a bonus from the pharmaceutical company; beyond the financial implications, this could also be detrimental to the patient’s health (Rabecs, 2005)

^f)^ A physician typically bills prenatal visits under CPT code 81002 (non-automated, non-microscopic urinalysis); as a service in a prenatal or postpartum visit that was included in the code of “global maternity service” and another bill for maternal care and delivery of a baby (Phillipsen et al., 2008)

^g)^ A patient complained that he went to the office and was given “an injection.” His insurance company received a bill for outpatient surgical care (USD 360). In another case, a pediatric nurse and her collaborating doctor billed for visits to the office of the parents and siblings of a child who was brought to the office due to illness. (It seemed they found both the disease and the billing to be contagious!). Neither parent had a medical history, nor did the siblings record visits or diagnoses (Phillipsen et al., 2008), and billing for advanced life support services when essential life support was provided (Barrett, 2006)

^h)^ The fee-for-service model means that physicians seek to maximize the number of services, which means maximizing their payment (Hennig-Schmidt et al., 2011); Another case, billing amounts of drugs that are higher than those dispensed; or billing for brand name drugs when less expensive generic versions are dispensed (Barrett, 2006). The ‘rolling labs’ administer tests provided by health care providers who temporarily visit shopping centres or nursing homes; these tests are simple but are billed as expensive tests (Borca, 2001)

^i)^ A physician billed for a fetal resting test (professional services using modifier 26) performed in the labour room of a local hospital by a nurse, who communicated the results to the physician, and the patient was discharged. The physician wrote no interpretation, nor was it filed in the patient’s medical record (Phillipsen et al., 2008)

^j)^ Double/duplicate billing and reimbursement acceptance from more than one payer source for the same service (Dolan & Farmer, 2016)

^k)^ Patient was admitted on January 22, 2016, and discharged on January 24 of the same year, with a diagnosis of tuberculosis and liver cirrhosis; the patient was readmitted on January 27, 2016, and discharged on January 29 of that year with the same diagnosis. Consequently, this case is classified as suspicious (Palutturi et al., 2019)

^l)^ Many patients have been treated not according to their class I or class II coverage. Therefore, the patient is treated at a lower level (Palutturi et al., 2019)

^m)^ Billing for drugs, procedures or services previously planned but later cancelled is rare, but possible fraud of this claim (Palutturi et al., 2019)

^n)^ A patient can make claims based on a diagnosis that is not real (Ogunbanjo & van Bogaert, 2014)

^o)^ One person obtained blank prescriptions from an office and then scanned them into a computer along with a genuine doctor’s signature, then used the prescriptions to generate high-cost drugs (Mundy & Chadwick, 2002)

^p)^ A Covington, Louisiana, couple and their company pleaded guilty to their roles in a scheme to create, market, and operate a fraudulent medical reimbursement program that defrauded the IRS and program participants out of more than $48 million (USAO-EDLA, 2019)

^q)^ Insured consumers can take advantage of an accident or illness by exaggerating the amount of the loss claimed or by filing fictitious claims (Ribeiro et al., 2020)

^r)^ A person without health coverage assumes a person’s identity with insurance coverage to obtain services, consultations, procedures, diagnostic support exams (Plomp & Grijpink, 2011)

^s)^ A patient can easily visit multiple doctors for prescriptions (often multiple times) (Thornton et al., 2015)

^t)^ Falsify employment/eligibility records to obtain a lower premium rate (Liu & Vasarhelyi, 2013)

^u)^ Three examples: i) An insurance agent, try to sell insurance directly to a person; typically, only the employer can contract. ii) the plan is not licensed in your state, and the agent (falsely) assures you that federal ERISA law exempts the plan from state licensing. iii) the plan looks like insurance, but the agent avoids calling it “insurance” and instead uses evasive terms like “benefits” (Thornton et al., 2015)

^v)^ A third-party administrator who processes claims on behalf of Medicare signed an integrity agreement with the Department of Justice in response to a number of allegations, including the fact that he made incorrect payments for claim filings (Liu & Vasarhelyi, 2013)

### RQ3: health insurance fraud factors

In this study, we define the factors as elements that can originate or influence the HIF. They can be categorized in multiple ways, such as by actors: internal staff, patient, intermediary and insurer (Yusuf, 2010); and by environment: context, organization and individual (Vahdati & Yasini, 2015; Vian, 2020), or internal and external (Akomea-Frimpong et al., 2016). These categorizations do not consider the relationship between each category, although factors influence more than one category, as in the doctor-patient interaction. Therefore, we added a new category called the collaborative environment, which includes interaction factors between different categories, either classified by actor type, or environment. For the presentation of the factors related to the HIF, we considered the macroenvironment categories if the factors are motivated by external influences (Lesch & Baker, 2013); mesoenvironment, if the factors are inspired by the context of the organizations; microenvironment, if the factors are associated with demographic and individual characteristics; and the collaborative environment.

We identified 47 factors that influence the occurrence of health insurance fraud, categorized into macroenvironment (6), mesoenvironment (15), microenvironment (20) and collaborative (6). For each study, we denoted with a positive sign (+) when the factor increased the HIF, and a negative sign (−) if the factor reduced the HIF; when used a single sign, it indicated that the study proved a theoretical or narrative contribution. A factor can show both signs simultaneously (+−), which means that its influence is ambivalent. In contrast, a double sign indicated that the study had an applied validation based on a method de experimentation or quasi experimentation.

#### Macroenvironment factors

A total of 14 studies explain 6 factors, 8 studies refer to norms and regulations, 3 studies to economic, political and social issues, and 3 studies to cultural issues. We can observed that some applied studies involve more than one factor. Culture is the only factor that contributes to increasing HIF (Zourrig et al., 2018); rules and regulations (Lesch & Baker, 2013) and geography (Manocchia et al., 2012) show an ambivalent influence, conditioned on the environment in which they were studied. At the theoretical level, it is found that complexity, infrastructure and economic, political and social conditions influence the HIF.

#### Mesoenvironment factors

We identified 26 studies that explained 15 factors, the most referenced factors are audit, supervision and control, with 8 studies, while 6 studies explain the general characteristics of the provider. The factors supported by applied studies that have shown influences in favour of the HIF occurring are the general characteristics of the provider (Herland et al., 2018; Kang et al., 2010), in favour of the HIF decrease (Vian, 2020), ambivalent (Massi et al., 2020); the management and policy of complaints show results that contribute to HIF (Vian, 2020) and ambivalent results (Lesch & Baker, 2013); while that the reputation shows ambivalent influence (Tseng & Kang, 2015), the audits, supervision and control contributes to reducing the HIF (Kang et al., 2010).

Each Macroenvironmental and Mesoenvironmental factor was briefly described, and one explanation was provided. We can observed that some studies involved more than one factor. For a better understanding, we present Table 3. In the next session, we analyze the studies according to the quality methodology proposed in this work to specify our findings in greater detail.

**Table 3:** Macroenvironmental and Mesoenvironmental factors that influence health insurance fraud

Description	Explanation	Contribution of studies					
		–	+−	+	–	++ − -	++
**Macroenvironmental Factors**							
Norms and regulations	There are increasingly stricter regulations to control medical services; however, diversity also increases its complexity.	(Krause, 2013; Vian et al., 2012)	(Kose et al., 2015; Maroun & Solomon, 2014; Myckowiak, 2009; Wang, 2014)	(Ribeiro et al., 2020)		(Lesch & Baker, 2013)	
Economic, political and social conditions	Economic recessions and other political conditions can condition lobbies, corruption and facilitate fraud.	(Ribeiro et al., 2020)	(Perez & Wing, 2019; Wang, 2014)				
Infrastructure	New equipment and technologies make fraud methods more sophisticated and complex.			(Brooks et al., 2012)			
Culture	It determines the way of acting of the population and their way of interacting conditions their behaviour.	(Ribeiro et al., 2020)		(Brooks et al., 2012)			(Zourrig et al., 2018)
The complexity of health systems	The complexity of health systems and their particularities make management, prevention and detection efforts and strategies are complicated.			(Faux et al., 2019; Vian et al., 2012)			
Geography	Geographic data are helpful for the prevention and detection of fraud and abuse in health services.			(Musal, 2010)		(Manocchia et al., 2012)	
**Mesoenvironmental Factors**							
General characteristics of the provider	It includes their legal nature for profit or not, location, competitiveness index, services they provide, schedules, payment statistics, history of their production.		(Bauder et al., 2017; Herland et al., 2019)		(Wan & Shasky, 2012)	(Massi et al., 2020)	(Herland et al., 2018; Kang et al., 2010)
Responsibility of the provider	If the provider maintains responsible conduct in its administrative and medical actions.	(Kerschbamer & Sutter, 2017)					
Measures of the administrative authority	The guidelines given by health care authorities influence payment for fraud and abuse, including medical records.	(Jator & Hughley, 2014)	(Tseng & Kang, 2015)				
Internal mechanisms of discipline	In the organization, some mechanisms punish fraud or abuse in health services.	(Myckowiak, 2009)					
Payment method and contracts	An essential part of the contract between provider and financier where payment of an amount is agreed based on assuming health risk management includes fees, payment model and contracts.		(Kose et al., 2015; Park et al., 2016; Shin et al., 2012)				
The medical record	The power of the medical record can improve the diligence and mastery of the documentation, which allows talking through the record without having to say a word.	(Dolan & Farmer, 2016; Smith et al., 2013)	(Gasquoine & Jordan, 2009)				
Audit, supervision, sanction and control	The design of practical audit and control strategies and programmes can improve the efficiency of providing services to patients and mitigating fraud, abuse or corruption. The penalty and fear of penalty are also considered in this factor.	(Hillerman et al., 2017; Maroun & Solomon, 2014; Myckowiak, 2009; Smith et al., 2013; Vian et al., 2012)	(Bourgeon & Picard, 2014; Dionne et al., 2009)		(Kang et al., 2010)		
Performance and quality evaluation system	An adequate system contributes to the quality of decision-making, feedback, dependence on employees and minimizes the possibility of fraud.	(Kerschbamer & Sutter, 2017)					
Reputation	Opinion, idea or concept that people have about a health service provider.		(Kerschbamer & Sutter, 2017)			(Tseng & Kang, 2015)	
Commercial implication	Medical practice is being bypassed by commercial considerations that could overlook fraud.	(Konijn et al., 2015)					
Lack of complaints management and policy	The complex nature of administrative, financial and benefits management and its *case-mix* condition the first line of claims management.	(Lee et al., 2016)				(Lesch & Baker, 2013)	(Wan & Shasky, 2012)
Reimbursement processes and billing characteristics	The billing pattern of the providers, including the duration of medical procedures or treatment of complex medical conditions.		(Lee et al., 2016)	(Hillerman et al., 2017; Kerschbamer & Sutter, 2017)			
Employability and job satisfaction	How satisfied employees are decreases staff turnover, absenteeism, motivation with their work and decrease corruption.			(Brooks et al., 2012)			
Patient identification mechanisms	Politics and identification procedures, including biometrics.	(Jator & Hughley, 2014)					
Types of health professionals	Health professionals are effective in controlling fraud in medical care.				(Goel, 2020)		

For each study, we denote with a positive sign (+) when the factor increases the HIF, and a negative sign (−) if the factor reduces the HIF; when used a single sign, it indicates that the study proved a theoretical or narrative contribution. A factor can show both signs simultaneously (+−), which means that its influence is ambivalent. In contrast, a double sign indicates that the study has an applied validation based on a method de experimentation or quasi experimentation

#### Microenvironment factors

A total of 35 studies contributed to explaining 20 factors of the microenvironment (see Table 4). Applied studies show that the two most referenced factors are prescription medications, and ethics and morals, both with 11 studies each. Other relevant factors are those related to demographic characteristics, among which sex and age stand out, with 4 studies each. In a study conducted in the state of Florida, USA, we found statistically significant results that encourage HIF: the western region, being a woman, being white, having health insurance, predominantly English language, having a condition sensitive to health, greater future risk of illness, health condition (Manocchia et al., 2012). Another factor that positively influences HIF is the prescription, dispensing, cost and consumption of medications (Aral et al., 2012; Herland et al., 2018; Lin et al., 2008; Liou et al., 2008; Weiss et al., 2015). In addition, factors related to users’ perceptions of health services such as inequity, injustice (Lesch & Baker, 2013), high deductibles, and coinsurance (Lammers & Schiller, 2010) can condition the HIF. Regarding the attitudes of claims adjusters, their decision is fickle, and has been demonstrated experimentally ambiguous (Tseng & Kang, 2015). Finally, the values that regulate human behaviour, such as ethics and morals, determine fraud.

**Table 4:** Microenvironmental and Collaborative factors that influence health insurance fraud

Description	Explanation	Contribution of studies					
		–	+−	+	–	++ − -	++
**Microenvironmental Factors**							
Demographic characteristics	Sex woman			(Musal, 2010; Zhou et al., 2016)		(Lesch & Baker, 2013)	(Manocchia et al., 2012)
	It affects more adults and the eldery			(Timofeyev & Busalaeva, 2019; Zhou et al., 2016)		(Lesch & Baker, 2013)	(Goel, 2020)
	Predominant white race						(Manocchia et al., 2012)
	Married, marital status		(Zhou et al., 2016)				
	Place of residence: more urbanized states			(Musal, 2010; Ribeiro et al., 2020)			(Goel, 2020)
	They have insured						(Manocchia et al., 2012)
Predominant English language	In cities where multiple languages are spoken, the predominant language can influence HIF.						(Manocchia et al., 2012)
Diagnostics	The diagnosis of patients is one of the main data produced in health providers, which is used to prevent and detect fraud and abuse and determine the future risk of becoming ill.	(Sun et al., 2020)		(Manocchia et al., 2012; Shin et al., 2012)		(Johnson & Nagarur, 2016; Massi et al., 2020; Park et al., 2016)	(Liou et al., 2008)
Medical and surgical treatments	Medical procedures, treatment and surgical decisions can become complex and specialized, which could hide fraud.			(Hillerman et al., 2017)		(Lee et al., 2020)	(Liou et al., 2008; Manocchia et al., 2012)
Specialities	Medical or other health -related specialties.		(Bauder et al., 2017)	(Shin et al., 2012)		(Herland et al., 2020; Johnson & Nagarur, 2016)	
Medications	Medical prescription, dispensing, cost and consumption are variables of analysis that can condition collusion or other forms of fraud and medical abuse.	(Haddad Soleymani et al., 2018; Sun et al., 2020; Victorri-Vigneau et al., 2009)		(Kose et al., 2015; Shin et al., 2012)		(Johnson & Nagarur, 2016)	(Aral et al., 2012; Herland et al., 2018; Lin et al., 2008; Liou et al., 2008; Weiss et al., 2015)
Chronic health condition	The health condition could condition the fraud, includes rare and orphan chronic conditions.				(Manocchia et al., 2012)		(Liou et al., 2008)
Risk of illness	The risk score for illness is considered.						(Manocchia et al., 2012)
Ethics and morals	The health insurance market is not immune to intrapersonal processes such as ethics and the offer of moral risks, which could influence the actors’ positions and their behaviour.	(Tseng, 2016)	(Bourgeon & Picard, 2014; Dionne et al., 2009; Jou & Hebenton, 2007; Kumar et al., 2011; Tseng & Kang, 2015; Wang, 2014; Zhou et al., 2016)	(Ribeiro et al., 2020)	(Duszak & Duszak, 2011)	(Lesch & Baker, 2013)	
Perception of inequity and injustice	The relational dynamic produces a perception of injustice or inequity.	(Ribeiro et al., 2020)				(Lesch & Baker, 2013)	
Information asymmetry	Asymmetric information occurs when one of the actors does not have the same information, this is reflected in the behaviours adopted by the different actors and their billing processes.			(Kerschbamer & Sutter, 2017; Kumar et al., 2011; Ribeiro et al., 2020; Zhou et al., 2016)			
The decision of the adjusters	The person who can decide when a settlement is approved or disapproved is one of the most decisive factors.					(Tseng & Kang, 2015)	
Strengthening of capacities	Training, seminars.	(Myckowiak, 2009)					
High deductibles and coinsurance	The deductible is a fixed payment before the insurance covering the remaining eligible expenses, while coinsurance is a percentage of the cost of care. Both are out-of-pocket payments and depend on the health insurance plan contract.						(Lammers & Schiller, 2010)
Bad economic situation	A bad economic situation generates financial pressure, which can cause fraud.			(Ribeiro et al., 2020)			
**Collaborative factors**							
Relationship between the health professional and the patient	The familiarity that exists between the health professional and the patient can influence fraud and abuse.		(Wang et al., 2017)	(Wan & Shasky, 2012)			
The complicity between the provider and the insurer	The rates agreed between providers and insurers are usually high to increase premiums, deductibles and coinsurance.			(Bayerstadler et al., 2016)			(Lin et al., 2008)
Relationship between the consumer and the provider	Relationships between consumers and providers could generate an excessive demand for health services. The high number of patients per provider could hide the possibility of HIF.			(Musal, 2010; Shin et al., 2012)			(Lin et al., 2008)
Relationship between the consumer and the insurer	Greater interaction between the consumer and the insurer through complaints or calls is associated with the fraud.					(Lesch & Baker, 2013)	(Manocchia et al., 2012)
The influence of bosses	The bosses often influence the personnel’s decisions to modify their behaviour, for example, the processes related to the approval of medical claims.						(Tseng & Kang, 2015)
The *guānxi* between insurance salespeople and customers	*Guānxi* refers to the lasting social connections and relationships that a person in China uses to exchange favours with a specific purpose. These connections can be expressed in attitudes, intentions or perceptions.						(Tseng, 2016)

For each study, we denote with a positive sign (+) when the factor increases the HIF, and a negative sign (−) if the factor reduces the HIF; when used a single sign, it indicates that the study proved a theoretical or narrative contribution. A factor can show both signs simultaneously (+−), which means that its influence is ambivalent. In contrast, a double sign indicates that the study has an applied validation based on a method de experimentation or quasi experimentation

#### Collaborative factors

A total of 10 studies contributed to explaining 6 collaborative factors (see Table 4), in which the most referenced factor was the relationship between the provider and the patient, with 3 studies. Relationships between the consumer provider, provider insurer (Lin et al., 2008), consumer insurer (Manocchia et al., 2012), the influence of bosses (Tseng & Kang, 2015) and *gGuānxi* (Tseng, 2016) encourage the increase in HIF.

We have proceeded to an analysis of the 47 identified factors, their corresponding influences supported by theoretical or applied contributions to increasing or decreasing the HIF (Tables 3 and 4). We also considered evaluating the quality of the 53 studies (Additional file 4) that support our RQ3 question (Fig. 1). For a better presentation, we categorized the studies by their quality score: high (11 to 13), medium (7 to 10) and low (4 to 6); the results are presented in Table 5.

**Table 5:** Studies that identify factors according to their quality assessment

	Quality of Studies															
	High [11–13]						Medium[7–10]						Low[4–6]			
Factors Contributions of studies	–	+−	+	–	++ − -	++	–	+−	+	–	++ − -	++	–	+−	+	++ − -
**Macroenvironmental Factors**																
1. Norms and regulations	--------------●--------------						------------------------●----			--------------●--------------			--●●●-----●●-------------			
2. Economic, political and social conditions	--------------●--------------						----●------------------------						--------------●--------------			
3. Infrastructure													------------------------●----			
4. Culture							----●------------------------			------------------------●----			------------------------●----			
5. The complexity of health systems													-----------------------●●---			
6. Geography	------------------------●----									--------------●--------------						
**Mesoenvironmental Factors**																
7. General characteristics of the provider	-------------●●-------------			----●--------●-------●●---												
8. Responsibility of the provider													----●------------------------			
9. Measures of the administrative authority	--------------●--------------												----●------------------------			
10. Internal mechanisms of discipline													----●------------------------			
11. Payment method and contracts	------------●●●------------															
12. The medical record							----●------------------------						----●--------●--------------			
13. Audit, supervision, sanction and control	----●--------●--------------			----●------------------------			----●------------------------						--●●●------●--------------			
14. Performance and quality evaluation system													----●------------------------			
15. Reputation				--------------●--------------									--------------●--------------			
16. Commercial implication	----●------------------------															
17. Lacks of Complaints management and policy	----●------------------------			------------------------●----						--------------●--------------						
18. Reimbursement processes and billing characteristics	--------------●--------●----												------------------------●----			
19. Employability and job satisfaction													------------------------●----			
20. Patient identification mechanisms													----●------------------------			
21. Types of health professionals (nurse)				----●------------------------												
**Microenvironmental Factors**																
22. Sex	------------------------●----						------------------------●----			--------------●--------●----						
23. Age				------------------------●----			-----------------------●●---			--------------●--------------						
24. Predominant race										------------------------●----						
25. Marital status							--------------●--------------									
26. Place of residence	------------------------●----			------------------------●----			------------------------●----									
27. They have insured status										------------------------●----						
28. Predominant language										------------------------●----						
29. Diagnostics	----●------------------●----			------------●●●------●----			------------------------●----									
30. Medical and surgical treatments	------------------------●----			------------------------●----						------------------------●----						---------●---------
31. Specialities	--------------●--------●----			-------------●●-------------												
32. Medications	--●●●---------------●●---			--------------●----●●●●●												
33. Chronic health condition				------------------------●----						----●------------------------						
34. Future risk of illness										------------------------●----						
35. Ethics and morals	-------------●●-------------						----●-------●●-------●----			----●--------●--------------			------------●●●------------			
36. Perception of inequity and injustice							----●------------------------			--------------●--------------						
37. Information asymmetry							----------------------●●●--						------------------------●----			
38. The decision of the adjusters				--------------●--------------												
39. Strengthening of capacities													----●------------------------			
40. Deductibles and coinsurance				------------------------●----												
41. Economic situation							------------------------●----									
**Collaborative factors**																
42. Relationship between the health professional and the patient	--------------●--------●----															
43. The complicity between the provider and the insurer	------------------------●----			------------------------●----												
44. Relationship between the consumer and the provider	-----------------------●●---			------------------------●----												
45. Relationship between the consumer and the insurer										--------------●--------●----						
46. The influence of bosses				------------------------●----												
47. The guānxi between insurance salespeople and customers										------------------------●----						

For each study (●), we denote with a positive sign (+) when the factor increases the HIF, and a negative sign (−) if the factor reduces the HIF; when used a single sign, it indicates that the study proved a theoretical or narrative contribution. A factor can show both signs simultaneously (+−), which means that its influence is ambivalent. In contrast, a double sign indicates that the study has an applied validation based on a method de experimentation or quasi experimentation

We confirmed that the factors are affected by other factors and depend on their studied and developed context. In the macroenvironment, in the category of high quality, concerning the factors, no study showed the influence on the HIF, while the geography presented theoretical influence in favour of the HIF, supported by a theoretical study. On the other hand, if we analyzed the category of medium quality, culture contributes to an increase in the fraud supported by an applied study (Zourrig et al., 2018), even when a theoretical study shows that it decreases the HIF.

In the mesoenvironment, in the high-quality category, the factors related to the audit, supervision, sanction, control (Hillerman et al., 2017; Maroun & Solomon, 2014; Myckowiak, 2009; Smith et al., 2013; Vian et al., 2012), and the type of health professional, particularly the nurses (Goel, 2020), shows influence in reducing HIF. Additionally, the general characteristics of the provider contribute to an increase in the HIF supported by two applied studies (Herland et al., 2018; Kang et al., 2010); even though a theoretical study shows that the HIF decreases, this study also confirms that the lack of policies and management of complaints increases fraud (Wan & Shasky, 2012). In this category, other factors (medical record, provider responsibility, provider internal mechanisms, internal staff evaluations, patient identification mechanisms, among others) have been shown to contribute to reducing HIF; several are theoretical or of medium or low quality.

In the microenvironment, in the high-quality category, no applied studies have demonstrated an influence that reduces HIF. While the factors related to having an older age to be deceived (> 65), place of residence (Goel, 2020), patient diagnoses, medical and surgical treatments (Liou et al., 2008), medications (Aral et al., 2012; Herland et al., 2018; Lin et al., 2008; Liou et al., 2008; Weiss et al., 2015), chronic health condition (Liou et al., 2008), and deductibles and coinsurance (Lammers & Schiller, 2010) showed influence in increasing the HIF. However, other theoretical studies showed that the diagnoses (Sun et al., 2020) and medications reduced HIF (Haddad Soleymani et al., 2018; Sun et al., 2020; Victorri-Vigneau et al., 2009). It is important to mention the factor related to ethics and morals, which has shown a theoretical and applied contribution to reducing the HIF.

In the high-quality category of collaborative environment, the provider-insurer relationship (Lin et al., 2008), the consumer-provider relationship (Lin et al., 2008), and bosses influence (Tseng & Kang, 2015); increased HIF. Also, in the middle-quality category, the consumer-insurer relationship (Manocchia et al., 2012), the *Guānx*i (Tseng, 2016), showed influence in increasing the HIF.

## Discussion

We analyzed 67 primary studies from January 2006 to July 2020, including definitions, manifestations, and HIF factors. Our findings identified 6 theoretical definitions, 22 manifestations (13 of the provider, 7 of the insurer and 2 of the insurer) and 47 factors categorized into macroenvironment (6), mesoenvironment (15), microenvironment (20), and collaborative (6).

### Definition fraud

The HIF definition that we have found is related to the general definition of insurance fraud given by the International Association of Insurance Supervisors (IAIS), which defines fraud as an act intended to obtain a dishonest advantage (IAIS, 2011). Similarly, the nongovernmental organization Transparency International defines fraud as a criminal or civil crime that consists of intentionally deceiving someone to obtain an unfair or illegal advantage (financial, political or otherwise) (Transparency International, 2017). In this same sense, other authors determine fraud as a criminal deception to generate illegal financial gains (Steinmeier, 2016). Likewise, Onwubiko specifies the concept of fraud as a criminal offence, illegal, intentional, deliberate act, characterized by deception, concealment, violation of trust or the use of dishonest means, which can cause injury or material damage (Onwubiko, 2020). In addition, our definition of HIF includes the one given by Yusuf et al.: «... is the one that covers the payments of benefits as a result of an illness or injury, includes insurance for accidental losses, medical expenses, disability or accidental death and dismemberment» (Yusuf & Ajemunigbohun, 2015). The HIS also identifies an institution or programme that helps pay for medical expenses, either through privately acquired health insurance, social insurance or a social welfare programme funded by the government (Brooks et al., 2017).

With the HIF definition, we seek to dispel the omission of a standard definition and highlight their element variability, which will potentially contribute to better identifying the characteristics of fraud, its causes (factors), manifestations, consequences and delimiting the differences with abuse, and the error. In general, the term abuse is used to define the practices of providers that result in unnecessary costs; these activities do not constitute fraud because they are legal (Gasquoine & Jordan, 2009). Likewise, the error is not intentional, so it does not constitute fraud (Brooks et al., 2017). These differences make the identification of the HIF more complex.

However, legal frameworks refer to fraud as a “conceptual swamp” (Green, 2007). The definition of HIF depends on the legal framework and regulations of the country or each state, so the same practices could be legal or illegal depending on their regulations. In the USA, the HIF is highly regulated with laws such as the Federal False Claims Act statute (31 USC 3279) - FCA and the Anti-Kickback Statute (42 USC 1320 a - 7) AKS., the Physician Self-referral Law (42 USC 1395nn)-StarK, the Health Insurance Portability and Accountability ACT of 1996-HIPAA (Myckowiak, 2009), while in other countries they lack regulation; so it could not be constituted in HIF.

The HIF definition is made tangible through its manifestations, which have been grouped into three categories: provider, underwriter, and insurer. In any category, the demonstration must meet the HIF definition and show intentionality on the perpetrator’s part to obtain some benefit. The act of defrauding is conscious and is evidenced day by day by self-referring patients, providing unnecessary care or misrepresenting clinical and administrative documents. However, when the evidence, they often argue deficiencies in supplies, medical materials, stock, in the number of professionals or sustain that the equipment is damaged or under maintenance, thereby circumventing the true intentions that they have as perpetrators.

### Manifestation of fraud

The most frequently studied manifestations are related to the provider, like ghost claims, the manipulation of documents and unnecessary care, where deception and intentional misrepresentation are the most evident. While in other manifestations such as coding, billing for services provided by unskilled personnel, and duplication of billing. They are more complex to detect and separate the error intentionality. At this point, experts must make strenuous efforts to develop cost-effective tools or strategies to separate and recognise the provider’s intent.

On the other hand, we appreciate that some manifestations lack studies, such as self-reference, a widespread manifestation and public knowledge, becoming even a common practice in HS. This phenomenon is explained by having a culture permissive to fraud, where ethics and morals are often bypassed by economic interests, the lack of patients to report and lax legislation. From the HIF manifestations of the subscriber, we found identity fraud more frequently, the one that takes advantage of the absence or inefficiency of the controls, and the one that clearly shows intentionality and deception. However, there is little evidence of the HIF manifestation defined as “Doctor Shopping” since it is confused with abuse, which will depend on regulation. A characteristic of the manifestations of the insurers' HIF is that they do not make the fraud visible since the organisations prefer to separate the fraudster silently protecting the reputational risk.

### Factors fraud

Regarding the factors, we have identified that they can increase or decrease the HIF. Moreover, these factors are interrelated in various ways, constituting a complex network whose behaviour over time could, to some extent, be unpredictable, contradictory and ambiguous. According to Brugé et al., «... the main problem lies in contrast between the simplicity of our administrations and the complexity of the problems to be solved; the classical administrative modus operandi consists of simplifying problems by reducing themselves to a specialized field...»; This explains why our results show the same factor with ambivalent influence, which confirms the sensitivity of the factors (Brugué et al., 2015).

The results in Table 5 compared Tables 3 and 4, considering with the quality assessment of the studies 5; in order to verify whether the initially found results maintain an influence on the HIF after considering only high and medium quality studies and excluding ambivalent results; and we find within the macroenvironment factors that norms and regulations confirm an increase in fraud (Ribeiro et al., 2020) as well as geography (Musal, 2010). However, the economy, politics and social conditions confirm a decrease in fraud (Ribeiro et al., 2020). On the other hand, the infrastructure (Brooks et al., 2012), the complexity of the health systems (Vian et al., 2012) (Faux et al., 2019) lose influence in favour of increasing the HIF. All this, supported by theoretical studies. Furthermore, with mixed results, we found that culture maintains the results concerning HIF (Ribeiro et al., 2020; Zourrig et al., 2018).

Likewise, by continuing with the verification of the results of the mesoenvironmental factors, considering only high and medium quality studies, we analyzed that the general characteristics of the provider, supported by applied studies, confirm the ambivalent results (Herland et al., 2018; Kang et al., 2010; Wan & Shasky, 2012). The Payment method and contracts also show ambivalent results and lose their influence on the HIF. The responsibility of the provider (Kerschbamer & Sutter, 2017), the measures of the administrative authority (Jator & Hughley, 2014), and the internal mechanisms of discipline (Myckowiak, 2009), the performance and quality evaluation system (Kerschbamer & Sutter, 2017), the commercial implication (Konijn et al., 2015), the employability and satisfaction with his work (Brooks et al., 2012), patient identification mechanisms (Jator & Hughley, 2014); they lose their influence in favour of reducing fraud, all supported by theoretical studies. Reputation only shows ambivalent results and loses its influence. Additionally, reimbursement processes and billing characteristics (Hillerman et al., 2017) confirm an increase in the HIF. Also, the Lacks of Complaints management and policy confirms the increase to HIF (Wan & Shasky, 2012) even when another theoretical study opposes this statement (Lee et al., 2016). An important finding is the audit, supervision, sanction and control, supported by an applied study (Kang et al., 2010) and two theoretical studies (Hillerman et al., 2017; Smith et al., 2013). The role of nurses, supported by an applied study (Goel, 2020), confirms their contribution to reducing fraud. Finally, the medical record supported by a theoretical study (Smith et al., 2013) confirms a decrease in HIF.

When interpreting the results of the microenvironment factors, we verify that an applied study confirms that being a women, and the risk of becoming ill, increase the probability of fraud (Manocchia et al., 2012); it is worth mentioning that the proportion of women who seek medical attention is higher than men. Likewise, an applied study confirms that the involvement of adults over 65 years of age increases the probability of fraud (Goel, 2020), and other theoretical studies confirm this (Timofeyev & Busalaeva, 2019; Zhou et al., 2016). As well as, being married maintains mixed results (Zhou et al., 2016) concerning HIF. In addition, the predominant white race (Manocchia et al., 2012), and the place of residence in more urbanized areas (Goel, 2020), confirm the increase in HIF based on applied studies; other theoretical studies confirm this finding (Musal, 2010; Ribeiro et al., 2020). Likewise, having health insurance, speaking an official language of the country based on an applied study increases the HIF (Manocchia et al., 2012). Also, the influence of diagnoses confirms an applied result that encourages HIF (Liou et al., 2008).

Two applied studies, and one theoretical study confirm that medical and surgical treatments, and chronic health condition increase the probability of fraud,  (Liou et al., 2008; Manocchia et al., 2012; Hillerman et al., 2017). In addition, the medical specialities and the bad economic situation confirms an increase in fraud, supported by an applied study (Shin et al., 2012). As well as, the influence of drugs confirms applied results that incentivize HIF (Aral et al., 2012; Herland et al., 2018; Lin et al., 2008; Liou et al., 2008; Weiss et al., 2015), Two applied studies confirm that medical and surgical treatments increase the probability of fraud (Liou et al., 2008; Manocchia et al., 2012). Another theoretical study confirms this claim (Hillerman et al., 2017). Additionally, the perception of inequity and injustice supported by a theoretical study (Ribeiro et al., 2020) and the asymmetry of information supported by three applied studies (Kumar et al., 2011; Ribeiro et al., 2020; Zhou et al., 2016) confirm a decrease in fraud. Also, for the decisions of the adjusters, the reputation only shows ambivalent results. Further, the capacity building shows results in favour of reducing HIF and loses influence. Likewise, the high deductibles and coinsurance, supported by an applied study, confirm the increase to the HIF (Lammers & Schiller, 2010). 

Regarding the collaborative environment, we have been able to verify that the relationship between the health professional and the patient confirms an increase in HIF (Shin et al., 2012). An applied study confirms a strong relationship between the provider and the insurer and that they increase the probability of fraud (Lin et al., 2008), and a theoretical study confirms this statement (Bayerstadler et al., 2016). An applied study confirms that a strong relationship between the consumer and the provider increases the probability of HIF (Lin et al., 2008). Two other theoretical studies confirm this statement (Musal, 2010; Shin et al., 2012). The consumer-insurer relationship, the influence of the bosses (Tseng & Kang, 2015), and Guānxi (Tseng, 2016) are support by applied studies, confirms the increase in HIF.

### Limitations

Regarding the limitations of our study, some deserve special attention. Regarding the design, we can mention the selection biases that did not include conferences, posters, editorials, letters, misprints and grey literature; however, we included the most relevant evidence from indexed journals. Likewise, another bias can be attributed to the limited search, that the range from January 1, 2006, to July 31, 2020, which it is justified by fraud penalty rules were given starting 2006. Despite the limitations, the study results reveal an effort to dispel the concept of HIF, recognize its manifestations and mainly identify its underlying factors, which can positively or negatively influence fraud. The results of this study may have important implications, as we seek to implement effective interventions that to date have eluded us.

## Conclusion

Regarding the definition of HIF that we propose is “an act based on deception or intentional misrepresentation to obtain illegal benefits concerning the coverage provided by health insurance”, which provides us with theoretical support that emphasizes its essential elements. We believe that this will distinguish it from abuse, corruption or error. The multifactorial nature of HIF is evident, as well as the particular characteristics of its manifestations, which are subject to its definition and may differ from one country to another according to its regulatory framework and the scope of health coverage provided. Identifying the factors and their influence will allow any subsequent attempt to propose practices that mitigate HIF.

The factors that have shown strength concerning reducing fraud are auditing, monitoring, sanction and control, nurses’ role (supported by applied studies), the economy, politics and social conditions, the medical record, and the commercial implication (supported by theoretical studies). On the other hand, the factors that have shown strength concerning increasing fraud are sex, age, predominant race, have health insurance, place of residence, medical and surgical treatments, chronic health conditions, risk of illness, deductibles and coinsurance, the complicity between the provider and the insurer, the relationship between the provider and the consumer, the relationship between the consumer and the insurer, the influence of the bosses and the Guanxi, (supported by applied studies), the geography, reimbursement processes and billing characteristics, information asymmetry, and poor economic situation of the patient (supported by theoretical studies).

Based on our results, we recommend that future investigations that explore HIF look for the relationships between the factors and their manifestations. Likewise, we suggest evaluating the relationship between the factors and the fraud theories themselves, developing computational methods that identify factors, identifying the costs generated and their impact on the HS, and proposing and implementing practices that mitigate the positive factors and enhance the negative ones. All with the purpose of help its detection and prevention with a comprehensive approach.

## Supplementary Information

**Additional file 1.** Scoping Reviews (PRISMA-ScR) Checklist. This file contains details of the Preferred Reporting Items for Systematic reviews and Meta-Analyses extension for Scoping Reviews (PRISMA-ScR) Checklist.

**Additional file 2.** Complete search strategy and characteristics of the included studies. This file contains details of the complete search strategy in the seven sources of information consulted.

**Additional file 3.** Excel matrix. This file contains details of all processed studies.

**Additional file 4.** Quality scoring of included studies. This file contains details of the four quality assessment factors for each of the 67 studies included in the analysis.

## Data Availability

The data supporting analysis of this work and additional files can be found in the main document.

## Abbreviations

ACM: Association for Computing Machinery
CPT: Current procedural terminology
E&M: Codes for Evaluation and Management
EconPapers: Economics working papers
ERISA: The Employee Retirement Income Security Act of 1974
HIF: Health insurance fraud
HIC: Health insurance coverage
HS: Health systems
IAIS: International Association of Insurance Supervisors
IRS: Internal Revenue Service, the federal government of the United States
MeSH: Medical Subject Headings
NHCAA: National Health Care Anti-Fraud Association
SJR: SCImago Journal Rank
TPA: A third-party administrator
USA: United States of America
WoS: Web of Science
