# Supplementary material for: Fourteen years of manifestations and factors of health insurance fraud, 2006–2020: a scoping review
Source: Health Justice. 2021 Sep 30;9:26. doi: 10.1186/s40352-021-00149-3 (PMC8482647; doi:10.1186/s40352-021-00149-3)
Supplement: Supplementary file 2 — Additional file 2. Complete search strategy and characteristics of the included studies. This file contains details of the complete search strategy in the seven sources of information consulted. [file 40352_2021_149_MOESM2_ESM.docx]

# **Additional file 2: Complete search strategy and characteristics of the included studies**

## **Query syntax used in the search**

Below we provide the reasons why we have proceeded to establish our search criteria, which are based on the need to define the fraud scenario in the health insurance environment. In this sense, the criteria that have been considered for the search were taken from the root terms (table additional 1):

a) MeSH (Unique ID: D005607: Fraud, healthcare)

b) MeSH (Unique ID: D007348: Health Insurance)

**Table additional 1.** String search

| **Source** | **Result count** | **Query Syntax** |
| --- | --- | --- |
| ACM | 251 | recordAbstract: ((healthcare OR provider OR insurance OR care OR assurance OR health OR medical) AND (bill OR claim OR prescription) AND (Fraud$ OR Abuse OR misrepresentation OR corrupt$ OR misconduct OR falsif$ OR forgery OR unbund$ OR kickback$ OR "fee splitting" OR "quackery counterfeit" OR bribing OR "up coding" OR whistleblowing OR decept$ OR theft OR crim$ OR misguide OR Kick?back$ OR kickback$ OR "excessive utili?ation" OR overutili?ation)) "filter": [Publication Date: (01/01/2006 TO 31/07/2020)]  Searched The ACM Guide to Computing Literature - Applied filters: Other periodicals - Since 2006 |
| EconPapers | 67 | (healthcare OR provider OR insurance OR care OR assurance OR health OR medical) AND (bill OR claim OR prescription) AND (Fraud$ OR Abuse OR misrepresentation OR corrupt$ OR misconduct OR falsif$ OR forgery OR unbund$ OR kickback$ OR "fee splitting" OR "quackery counterfeit" OR bribing OR "up coding" OR whistleblowing OR decept$ OR theft OR crim$ OR misguide OR Kick?back$ OR kickback$ OR "excessive utili?ation" OR overutili?ation) AND (-web -voip -rfid -computers -game -rdbms -wireless -smart -authentication -cryptography -biometrics -barcode -microfinance -legislature -bank$ -tax -credit -voting -urban -reform -internet -card -delivery -financial -automobile -sex$ -drug -child -obesity -substance -tobacco -pregnancy -prenatal -malpractice -epidemiology -rural -depression -diagnostic -chronic -experiment -food -hiv -aids -diabetic -fracture -oncology -pediatric -tramadol -alcohol -dent$ -suicide -racist -torture -malaria -vehicle -domesti$ -human$ -opioid) in articles |
| Pubmed | 44 | (fraud[Title/Abstract] OR Kickbacks[Title/Abstract] OR Kickback[Title/Abstract] OR "Drug Counterfeiting"[Title/Abstract] OR "Counterfeiting, Drug"[Title/Abstract] OR "Health Care Fraud"[Title/Abstract] OR "Healthcare Fraud"[Title/Abstract] OR "Fraud, Healthcare"[Title/Abstract] OR "Fraud, Health Care"[Title/Abstract] OR "Health Care Frauds"[Title/Abstract]) AND ("Health Insurance"[Title/Abstract] OR "Health Insurance, Voluntary"[Title/Abstract] OR "Insurance, Voluntary Health"[Title/Abstract] OR "Voluntary Health Insurance"[Title/Abstract] OR "Group Health Insurance"[Title/Abstract] OR "Health Insurance, Group"[Title/Abstract] OR "Insurance, Group Health"[Title/Abstract])  Filters applied: Journal Article and year 2006-2020 |
| ScienceDirect | 152 | Expert Search – Only Journal: (bill OR claim OR prescription) AND fraud  Title, abstract or author-specified keywords: (healthcare OR provider OR insurance OR care OR assurance OR health OR medical OR hospital) years 2006 - 2020 |
| Scopus | 95 | TITLE-ABS-KEY ( providers OR insurance OR care OR assurance OR health OR medical OR healthcare ) AND TITLE-ABS-KEY ( bill OR claim ) AND TITLE-ABS-KEY ( fraud OR fraud$ OR abuse OR misrepresentation OR corrupt$ OR misconduct OR falsif$ OR forgery OR unbund$ OR kickback OR "fee splitting" OR "quackery counterfeit" OR bribing OR "up coding" OR whistleblowing OR decept$ OR theft OR forgery OR crim$ OR misguide OR kick?back$ OR kickback$ OR "excessive utili?ation" OR overutili?ation ) AND NOT TITLE-ABS-KEY ( web OR voip OR rfid OR computers OR game OR rdbms OR wireless OR smart OR authentication OR cryptography OR biometrics OR barcode OR microfinance OR legislature OR bank$ OR tax OR credit OR voting OR urban OR reform OR internet OR card OR delivery OR financial OR automobile OR sex$ OR drug OR child OR obesity OR substance OR tobacco OR pregnancy OR prenatal OR epidemiology OR rural OR depression OR chronic OR food OR hiv OR aids OR diabetic OR fracture OR oncology OR pediatric OR tramadol OR alcohol OR dent$ OR suicide OR racist OR torture OR malaria OR vehicle OR domesti$ OR human$ OR opioid OR article ) AND ( LIMIT-TO ( SRCTYPE , "j" ) ) AND ( LIMIT-TO ( DOCTYPE , "ar" ) ) AND ( LIMIT-TO ( PUBYEAR , 2020 ) OR LIMIT-TO ( PUBYEAR , 2019 ) OR LIMIT-TO ( PUBYEAR , 2018 ) OR LIMIT-TO ( PUBYEAR , 2017 ) OR LIMIT-TO ( PUBYEAR , 2016 ) OR LIMIT-TO ( PUBYEAR , 2015 ) OR LIMIT-TO ( PUBYEAR , 2014 ) OR LIMIT-TO ( PUBYEAR , 2013 ) OR LIMIT-TO ( PUBYEAR , 2012 ) OR LIMIT-TO ( PUBYEAR , 2011 ) OR LIMIT-TO ( PUBYEAR , 2010 ) OR LIMIT-TO ( PUBYEAR , 2009 ) OR LIMIT-TO ( PUBYEAR , 2008 ) OR LIMIT-TO ( PUBYEAR , 2007 ) OR LIMIT-TO ( PUBYEAR , 2006 ) ) |
| Springer link | 277 | fraud* AND claim AND health* AND (fraud or abuse) AND NOT (voip OR rfid OR game OR microfinance OR legislature OR bank$ OR tax OR automobile OR tobacco OR tramadol OR alcohol OR depression OR food OR hiv OR aids OR suicide OR malaria OR vehicle OR opioid$ OR Traumatic OR Stress OR "child abuse" OR P2P OR overdose OR steroids OR cosmetic OR religiosity OR fertility OR plagiari* OR DNA OR oxitocin OR tumor OR "Peer Review")  within Article years: 2006 - 2020 |
| WoS | 58 | TEMA: ((fraud OR kickbacks OR kickback OR "Drug Counterfeiting" OR "Counterfeiting, Drug" OR "Health Care Fraud" OR "Healthcare Fraud" OR "Fraud, Healthcare" OR "Fraud, Health Care" OR "Health Care Frauds") AND ("Health Insurance" OR "Health Insurance, Voluntary" OR "Insurance, Voluntary Health" OR "Voluntary Health Insurance" OR "Group Health Insurance" OR "Health Insurance, Group" OR "Insurance, Group Health"))  TIPOS DE DOCUMENTOS: (ARTICLE OR EARLY ACCESS)  Período de tiempo: 2006-2020. Índices: SCI-EXPANDED, SSCI, A&HCI, ESCI. |
| **Total** | **944** |  |

## **Characteristics of the included studies**

We identified a total of 946 studies, including articles and other sources, and of these, 67 were included, representing 7.1%. The information sources PubMed, EconPapers, Science Direct, and Web of Science (WoS) were those that contained the highest volume of studies. In PubMed, 12 primary studies were identified to be included, as shown in table additional 2.

**Table additional 2.** Studies identified and included

| Source | Studies identified * | | Studies included ^†^ | |
| --- | --- | --- | --- | --- |
|  |  |  |  |  |
| ACM | 251 | 26,5 % | 7 | 10,5 % |
| EconPapers | 67 | 7,1 % | 10 | 14,9 % |
| PubMed | 44 | 4,7 % | 12 | 18,0 % |
| Science Direct | 152 | 16,1 % | 10 | 14,9 % |
| Scopus | 95 | 10,0 % | 8 | 11,9 % |
| Springer | 277 | 29,3 % | 8 | 11,9 % |
| WoS | 58 | 6,1 % | 10 | 14,9 % |
| Others | 2 | 0,2 % | 2 | 3,0 % |
| Total | 946 | 100 % | 67 | 100 % |

* Some studies appear in more than one source.

^†^ Repeated studies have been excluded, considering the alphabetical order of the data sources consulted.

## **Trend of included studies**

Of the included studies, we observed more publications to July 2020 with an upward trend (see Fig additional 1); the principal authors of the included studies, 47 8%, come from the United States of America and 25.3% from Asian countries; Likewise, considering the country of origin of the indexed journals, 65.7% come from Europe and 26.8% the United States of America.

**Fig Additional 1.** The trend of included studies

## **Studies included by sources of information**

Table additional 3 shows the included studies by sources of information, which provides answers to each research question defined in the eligibility criteria section.

**Table additional 3.** Studies included by research questions

| **Source** | **RQ1**  **Definition** | **RQ2**  **M*nifest*tion** | **RQ3**  **Influencing f*ctors** |
| --- | --- | --- | --- |
| **ACM** | [45]* | [45]*^,†^, [60]^†^, [64]^†^ | [16], [45]^†^, [60]^†^, [64]^†^, [102], [104], [106] |
| **EconPapers** |  | [7]^†^, [10], [17]^†^, [61]^†^, [65], [66]^†^ | [7]^†^, [17]^†^, [61]^†^, [66]^†^, [86], [89], [92], [103] |
| **PubMed** | [26] | [62]^†^, [68], [69]^†^ | [10], [21], [62]^†^, [69]^†^, [95], [100], [105], [110], [112], [115] |
| **ScienceDirect** | [46]* | [46]*^,†^, [50], [54]^†^, [55]^†^, [56]^†^ | [30], [46]^†^, [54]^†^, [55]^†^, [56]^†^, [90], [96], [101], [114] |
| **Scopus** | [44]* [47]* | [44]*^,†^, [47]*, [51], [52], [57] | [44]^†^, [87], [109], [113] |
| **Springer** |  | [23], [53]^†^, [58]^†^ | [53]^†^, [58]^†^, [97], [98], [88], [111], [107] |
| **WoS** |  | [63], [67]^†^, [70]^†^, [71]^†^, [72] | [67]^†^, [70]^†^, [71]^†^, [93], [91], [94], [99], [108] |
| **Others** | [43] | [59] |  |
| **Total** | **6** | **31** | **53** |

* Four studies covered theoretical definition and manifestation.

^†^ 19 studies covered manifestation and influencing factors.
