# Supplementary material for: Fourteen years of manifestations and factors of health insurance fraud, 2006–2020: a scoping review
Source: Health Justice. 2021 Sep 30;9:26. doi: 10.1186/s40352-021-00149-3 (PMC8482647; doi:10.1186/s40352-021-00149-3)
Supplement: Supplementary file 4 — Additional file 4. Quality scoring of included studies. This file contains details of the four quality assessment factors for each of the 67 studies included in the analysis. [file 40352_2021_149_MOESM4_ESM.docx]

**Additional file 4: Quality scoring of included studies**

To evaluate the quality and rigour of the studies, we used a tool for integrative reviews, which is based on four factors: type of study, sampling method, detail of the data collection method and analysis. The possible score generated by this tool varies between 4 (qualitative design, sampling and collection of unexplained data and narrative analysis) and 13 (quantitative experimental design, random sampling, explained data collection and inferential statistics)

Quality scoring of included studies:

| **N°** | **Reference** | **Study type ^a^** | **Sampling ^b^** | **Method**  **Detail ^c^** | **Analysis ^d^** | **Total Score** | **doi or URL** |
| --- | --- | --- | --- | --- | --- | --- | --- |
| 1 | Aral et al. (2012) [32] | 6 | 1 | 1 | 3 | 11 | 10.1016/j.cmpb.2011.09.003. |
| 2 | Baltussen et al. (2006) [47] | 4 | 1 | 1 | 2 | 8 | 10.1111/j.1365-3156.2006.01621.x. |
| 3 | Bauder & Khoshgoftaar (2017) [74] | 6 | 3 | 1 | 3 | 13 | 10.1007/s10742-017-0172-1. |
| 4 | Bauder & Khoshgoftaar (2020) [68] | 6 | 3 | 1 | 3 | 13 | 10.3233/IDA-184415. |
| 5 | Bayerstadler et al. (2016) [14] | 6 | 3 | 1 | 3 | 13 | 10.1016/j.insmatheco.2016.09.013. |
| 6 | Bourgeon & Picard (2014) [45] | 4 | 0 | 1 | 1 | 6 | 10.1257/aer.104.9.2900. |
| 7 | Brooks et al. (2012) [73] | 4 | 1 | 0 | 1 | 6 | 10.1057/sj.2011.7. |
| 8 | Dionne et al. (2009) [35] | 6 | 3 | 1 | 3 | 13 | 10.1287/mnsc.1080.0905. |
| 9 | Dolan & Farmer (2016) [55] | 3 | 0 | 0 | 1 | 4 | 10.1016/j.nurpra.2015.09.018. |
| 10 | Duszak & Duszak (2011) [63] | 4 | 3 | 1 | 2 | 10 | 10.1016/j.optm.2010.09.015. |
| 11 | Ekin et al. (2018) [40] | 6 | 1 | 1 | 3 | 11 | 10.1111/insr.12269. |
| 12 | Faux et al. (2019) [49] | 3 | 0 | 0 | 1 | 4 | <https://opus.lib.uts.edu.au/bitstream/10453/136958/4/j09_v027_JLM_pt01_Faux_Art%20%281%29%20final%20accepted.pdf> |
| 13 | Francis (2020) [56] | 3 | 0 | 0 | 1 | 4 | 10.1016/j.clindermatol.2020.02.009. |
| 14 | Gasquoine & Jordan (2009) [64] | 3 | 0 | 0 | 1 | 4 | 10.1037/a0013645. |
| 15 | Geruso & Rosen (2015) [83] | 4 | 1 | 1 | 3 | 9 | 10.1111/jori.12046. |
| 16 | Goel (2020) [82] | 6 | 1 | 1 | 3 | 11 | 10.1002/mde.3117. |
| 17 | Haddad et al. (2018) [53] | 6 | 3 | 1 | 3 | 13 | 10.1007/s40199-018-0227-z. |
| 18 | Herland et al. (2018) [76] | 6 | 3 | 1 | 3 | 13 | 10.1186/s40537-018-0138-3. |
| 19 | Herland et al. (2019) [75] | 6 | 3 | 1 | 3 | 13 | 10.1186/s40537-019-0181-8. |
| 20 | Herland et al. (2020) [77] | 6 | 3 | 1 | 3 | 13 | 10.1007/s10729-018-9460-8. |
| 21 | Hillerman et al. (2017) [62] | 6 | 3 | 1 | 3 | 13 | 10.1016/j.jocs.2017.02.007. |
| 22 | Jator & Hughley (2014) [81] | 3 | 0 | 0 | 1 | 4 | 10.1309/LMIEC52ZF7RLLURK. |
| 23 | Johnson & Nagarur (2016) [48] | 6 | 3 | 1 | 3 | 13 | 10.1007/s10729-015-9317-3. |
| 24 | Jou & Hebenton (2007) [57] | 3 | 1 | 0 | 1 | 5 | 10.1016/j.ijsl.2007.04.002. |
| 25 | Joudaki et al. (2015) [10] | 6 | 1 | 1 | 2 | 10 | 10.15171/ijhpm.2015.196. |
| 26 | Kang et al. (2010) [23] | 6 | 3 | 1 | 3 | 13 | 10.1016/j.healthpol.2009.10.003. |
| 27 | Kerschbamer & Sutter (2017) [87] | 3 | 0 | 0 | 1 | 4 | 10.1093/cesifo/ifx001. |
| 28 | Konijn et al. (2015) [36] | 6 | 3 | 1 | 3 | 13 | 10.1007/s10844-014-0313-8. |
| 29 | Kose et al. (2015) [6] | 6 | 3 | 1 | 3 | 13 | 10.1016/j.asoc.2015.07.018. |
| 30 | Krause (2013) [60] | 3 | 0 | 0 | 1 | 4 | 10.1378/chest.12-2889. |
| 31 | Kumar el al. (2011) [71] | 4 | 3 | 1 | 2 | 10 | 10.1177/097206341101300302. |
| 32 | Lammers & Schiller (2010) [78] | 6 | 3 | 1 | 3 | 13 | 10.1007/s12297-010-0097-z. |
| 33 | Lee et al. (2016) [59] | 3 | 0 | 0 | 1 | 4 | 10.1016/j.jom.2016.09.001. |
| 34 | Lee et al. (2020) [12] | 6 | 3 | 1 | 3 | 13 | 10.1016/j.jbi.2020.103423. |
| 35 | Lesch & Baker (2013) [42] | 4 | 1 | 1 | 3 | 9 | <http://www.insuranceissues.org/PDFs/361LB.pdf> |
| 36 | Li et al. (2008) [25] | 4 | 1 | 1 | 2 | 8 | 10.1007/s10729-007-9045-4. |
| 37 | Lin et al. (2008) [37] | 6 | 3 | 1 | 3 | 13 | 10.1016/j.eswa.2007.02.038. |
| 38 | Lin et al. (2009) [79] | 6 | 3 | 1 | 3 | 13 | 10.1111/j.1365-2753.2007.00940.x. |
| 39 | Liou et al. (2008) [51] | 6 | 3 | 1 | 3 | 13 | 10.1007/s10729-008-9054-y. |
| 40 | Manchikanti & Hirsch (2009) [9] | 3 | 0 | 0 | 2 | 5 | <https://www.painphysicianjournal.com/current/pdf?article=MTE5Mw%3D%3D&journal=48> |
| 41 | Manocchia et al. (2012) [39] | 4 | 1 | 1 | 3 | 9 | 10.1504/IJPP.2012.045878. |
| 42 | Maroun & Solomon (2014) [43] | 3 | 1 | 1 | 1 | 6 | 10.1016/j.accfor.2013.04.007. |
| 43 | Massi et al. (2020) [72] | 6 | 1 | 1 | 3 | 11 | 10.1186/s12911-020-01143-9. |
| 44 | Musal (2010) [61] | 6 | 3 | 1 | 3 | 13 | 10.1016/j.eswa.2010.06.095. |
| 45 | Myckowiak (2009) [84] | 3 | 0 | 0 | 1 | 4 | <https://www.painphysicianjournal.com/current/pdf?article=MTIzMQ%3D%3D&journal=49> |
| 46 | NHCAA (2018) [89] | 3 | 0 | 0 | 1 | 4 | <http://www.nhcaa.org/resources/health-care-anti-fraud-resources/the-challenge-of-health-care-fraud/> |
| 47 | Palutturi et al. (2019) [67] | 3 | 2 | 1 | 2 | 8 | <https://www.ijicc.net/images/vol8iss5/8509_Palutturi_2019_E_R.pdf> |
| 48 | Park et al. (2016) [50] | 6 | 3 | 1 | 3 | 13 | 10.3414/ME15-01-0076. |
| 49 | Perez & Wing (2019) [38] | 6 | 1 | 1 | 3 | 11 | 10.1162/ajhe_a_00130. |
| 50 | Phillipsen et al. (2008) [66] | 3 | 1 | 0 | 1 | 5 | 10.1016/j.nurpra.2008.09.006. |
| 51 | Ribeiro et al. (2020) [80] | 5 | 1 | 1 | 3 | 10 | 10.1007/s10611-019-09857-2. |
| 52 | Sheffali & Deepa (2019) [65] | 6 | 1 | 1 | 3 | 11 | 10.35940/ijitee.K1578.0881119. |
| 53 | Shin et al. (2012) [33] | 6 | 1 | 1 | 3 | 11 | 10.1016/j.eswa.2012.01.105. |
| 54 | Smith et al. (2013) [58] | 4 | 1 | 1 | 2 | 8 | 10.1331/JAPhA.2013.12165. |
| 55 | Sun et al. (2020) [52] | 6 | 3 | 1 | 3 | 13 | 10.2196/17653. |
| 56 | Timofeyev & Busalaeva (2019) [70] | 5 | 1 | 1 | 2 | 9 | 10.1057/s41284-019-00209-2. |
| 57 | Tseng (2016) [88] | 6 | 3 | 1 | 3 | 13 | 10.1108/IJCMA-08-2015-0049. |
| 58 | Tseng & Kang (2015) [44] | 4 | 2 | 1 | 3 | 10 | 10.1057/gpp.2014.39. |
| 59 | Vian et al. (2012) [85] | 3 | 0 | 0 | 1 | 4 | 10.1002/pad.1607. |
| 60 | Victorri-Vigneau et al. (2009) [46] | 6 | 3 | 1 | 2 | 12 | 10.2515/therapie/2009004. |
| 61 | Wan & Shasky (2012) [8] | 6 | 3 | 1 | 3 | 13 | 10.1504/IJPP.2012.045877. |
| 62 | Wang (2014) [86] | 3 | 0 | 0 | 1 | 4 | 10.3928/00485713-21040707-08. |
| 63 | Wang et al. (2017) [54] | 6 | 3 | 1 | 3 | 13 | 10.1016/j.artmed.2016.12.002. |
| 64 | Weiss et al. (2015) [34] | 6 | 3 | 1 | 3 | 13 | 10.1007/s10489-015-0685-7. |
| 65 | Yang (2003) [90] | 3 | 1 | 1 | 2 | 7 | [https://etd.lis.nsysu.edu.tw//ETD-db/ETD-search-c/view_etd?URN=etd-0612103-091248](https://etd.lis.nsysu.edu.tw/ETD-db/ETD-search-c/view_etd?URN=etd-0612103-091248) |
| 66 | Zhou et al. (2016) [41] | 4 | 1 | 1 | 3 | 9 | 10.1080/1540496X.2016.1177787. |
| 67 | Zourrig et al. (2018) [69] | 4 | 2 | 1 | 3 | 10 | 10.1108/IJQSS-02-2017-0016. |
|  | **Range** | **3-5** | **0-3** | **0-1** | **1-3** | **4-13** |  |
|  | **Mean** | **4.78** | **1.67** | **0.76** | **2.33** | **9.54** |  |

^a^ Study design scores: 3=qualitative design; 4=quantitative descriptive design; 5=mixed qualitative and quantitative descriptive; 6=quantitative experimental and quasi-experimental

^b^ Sampling (for primary study aim): 0=Not explained; 1=Convenience; 2=Purposive or Case matching/cohort; 3=Random or 100%

^c^ Method detail: 1=Methods and tools; 0=Not explained

^d^ Analysis: (highest level reported): 1=Narrative; 2=Descriptive statistics; 3=Inferential statistics.
